# Identification of a Novel Quinoxaline-Isoselenourea Targeting the STAT3 Pathway as a Potential Melanoma Therapeutic

**DOI:** 10.3390/ijms20030521

**Published:** 2019-01-26

**Authors:** Verónica Alcolea, Deepkamal N. Karelia, Manoj K. Pandey, Daniel Plano, Parvesh Singh, Juan Antonio Palop, Shantu Amin, Carmen Sanmartín, Arun K. Sharma

**Affiliations:** 1 Department of Pharmaceutical Technology and Chemistry, School of Pharmacy and Nutrition, University of Navarra, Irunlarrea 1, E-31008 Pamplona, Spain; valcolea@alumni.unav.es (V.A.); dplano@unav.es (D.P.); jpalop@unav.es (J.A.P.); sanmartin@unav.es (C.S.); 2 Instituto de Investigación Sanitaria de Navarra (IdiSNA), Irunlarrea 3, E-31008 Pamplona, Spain; 3 Department of Pharmacology, Penn State Cancer Institute, CH72, Penn State College of Medicine, 500 University Drive, Hershey, PA 17033, USA; dkarelia@pennstatehealth.psu.edu (D.N.K.); pandey@rowan.edu (M.K.P.); sga3@psu.edu (S.A.); 4 Department of Biomedical Sciences, Cooper Medical School of Rowan University, Camden, NJ 08103, USA; 5 School of Chemistry and Physics, University of Kwa-Zulu Natal (UKZN), Westville Campus, Durban 4000, South Africa; singhp4@ukzn.ac.za

**Keywords:** selenium, isoselenourea, melanoma, STAT3, apoptosis

## Abstract

The prognosis for patients with metastatic melanoma remains very poor. Constitutive signal transducer and activator of transcription 3 (STAT3) activation has been correlated to metastasis, poor patient survival, larger tumor size, and acquired resistance against vemurafenib (PLX-4032), suggesting its potential as a molecular target. We recently designed a series of isoseleno- and isothio-urea derivatives of several biologically active heterocyclic scaffolds. The cytotoxic effects of lead isoseleno- and isothio-urea derivatives (compounds 1 and 3) were studied in a panel of five melanoma cell lines, including B-RAF^V600E^-mutant and wild-type (WT) cells. Compound 1 (IC_50_ range 0.8–3.8 µM) showed lower IC_50_ values than compound 3 (IC_50_ range 8.1–38.7 µM) and the mutant B-RAF specific inhibitor PLX-4032 (IC_50_ ranging from 0.4 to >50 µM), especially at a short treatment time (24 h). These effects were long-lasting, since melanoma cells did not recover their proliferative potential after 14 days of treatment. In addition, we confirmed that compound 1 induced cell death by apoptosis using Live-and-Dead, Annexin V, and Caspase3/7 apoptosis assays. Furthermore, compound 1 reduced the protein levels of STAT3 and its phosphorylation, as well as decreased the expression of STAT3-regulated genes involved in metastasis and survival, such as survivin and c-myc. Compound 1 also upregulated the cell cycle inhibitor p21. Docking studies further revealed the favorable binding of compound 1 with the SH2 domain of STAT3, suggesting it acts through STAT3 inhibition. Taken together, our results suggest that compound 1 induces apoptosis by means of the inhibition of the STAT3 pathway, non-specifically targeting both B-RAF-mutant and WT melanoma cells, with much higher cytotoxicity than the current therapeutic drug PLX-4032.

## 1. Introduction

Melanoma is originated from the malignant transformation of melanocytes. Its incidence has been rapidly increasing, with a 5-fold increase in the last three decades [1]. The American Cancer Society’s estimates for melanoma in the United States for 2018 are: About 91,270 new melanomas will be diagnosed (about 55,150 in men and 36,120 in women) and about 9320 people are expected to die of melanoma (about 5990 men and 3330 women). Nearly 1,330,300 new cases of melanoma and about 126,000 deaths are estimated to occur worldwide in 2018. The regions more affected are those with white population, highest incidences being in Australia, New Zealand, Northern America, and Northern and Western Europe [2]. If diagnosed in early stages, melanoma can be easily removed by surgical excision but in metastatic form, it is one of the most aggressive malignancies with low survival rates [3].

The mitogen-activated protein kinase (MAPK) signaling cascade is a key pathway in melanoma survival and proliferation. Constitutive activation of B-RAF kinase due to B-RAF^V600E^ mutation occurs in nearly 50% of cutaneous melanomas [4]. Moreover, constitutive activation of signal transducer and activator of transcription 3 (STAT3) has been implicated in many oncogenic features, as it regulates the transcription of a variety of genes involved in cell proliferation, apoptosis, angiogenesis, and metastasis. The constitutive activation of STAT3 has been reported in melanoma patients [5]. The high expression of STAT3 has been correlated to large tumor diameter and depth, lymph node metastasis, high expression of MMP-2 and -9, and poor patient survival [6,7,8]. 

Traditional treatments for melanoma have shown low response rates and are associated with severe adverse effects. The introduction of agents that specifically target the MAPK pathway, such as vemurafenib (PLX-4032), dabrafenib, and trametinib, has significantly improved the overall survival of patients bearing the B-RAF^V600E^ mutation [9]. Although treatment with these inhibitors induces a quick initial response, the effect is not durable because of the rapid development of drug resistance [10]. The acquired resistance against vemurafenib is implicated in the activation of STAT3 and its signaling pathways [11]. Importantly, suppression of STAT3 activity disrupts B-RAF^V600E^-mediated induction of anti-apoptotic proteins and reduces melanoma cell survival [12]. In addition, these treatments are ineffective in patients without the B-RAF^V600E^ mutation [10]. Therefore, there is an urgent need to develop novel agents for melanoma treatment. 

In the last decade, selenium-containing compounds have emerged as promising anticancer agents due to their efficacy and selectivity [13]. Several studies, including those from our laboratories, have reported the role of different selenium-containing compounds in the prevention and treatment of melanoma, including its metastasis [14,15,16,17,18,19,20]. Of these, *Se,Se′*-1,4-phenylenebis(1,2-ethanediyl)bisisoselenourea (PBISe), an isoselenourea derivative, has been found to inhibit proliferation and promote apoptosis in melanoma cells in cell culture models [19]. It should be noted that PBISe was over 10-fold more effective than its isosteric sulfur analog *S*,*S*’-1,4-phenylenebis(1,2-ethanediyl)bisisothiourea (PBIT). Furthermore, the topical application of PBISe significantly delayed xenografted melanoma tumors growth [21]. 

In continuation of our pursuits towards developing novel organoselenium compounds as anticancer agents, we recently reported the synthesis and screening of a series of novel isoselenourea and corresponding isothiourea analogs [22]. This series consisted of new hybrid compounds containing an isoselenourea or an isothiourea group and different carbo- and hetero-cyclic scaffolds which have been traditionally included in anticancer agents [22]. Considering the encouraging results of PBISe in melanoma, we decided to evaluate the potential of the novel analogs as potential melanoma therapeutics. For this purpose, we selected an isoselenourea and an isothiourea derivative which showed the highest activity against a melanoma cell line (1205Lu) in our previous screening (compounds 1 and 3, respectively) [22]. The corresponding sulfur analog of compound 1, i.e., isothiourea compound 2, was also tested in order to determine if the compound selenium-containing 1 was more active than the sulfur compound 2 against melanoma cells, similar to what observed in the case of PBISe. This study examines the effects of these compounds on various melanoma cell lines and further evaluates the underlying mechanisms of action of compound 1.

## 2. Results

### 2.1. Synthesis of Compounds 1, 2, and 3

**Figure 1:**
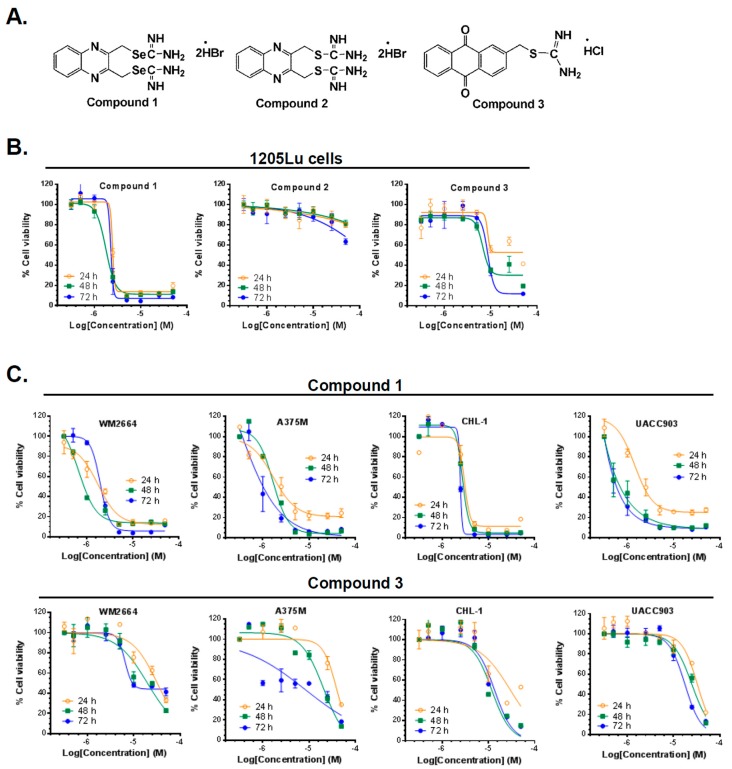
Compounds 1 and 3 were effective at reducing cell viability of different melanoma cancer cell lines. (**A**) Structures of the compounds, (**B**) Screening of the three agents in melanoma cells (1205Lu) for 24, 48, and 72 h using the MTT (cell viability) assay (**C**) Cell viability (MTT) results for compounds 1 and 3 in four different melanoma cancer cell lines at three different time points. Graphs were obtained by performing non-linear regression analysis using variable slope. Error bars represent mean ± SD.

The structures of quinoxaline-2,3-diylbis(methylene)dicarbamimidoselenoate dihydrobromide (compound 1), its sulfur analog quinoxaline-2,3-diylbis(methylene)dicarbamimidothioate dihydrobromide (compound 2), and (9,10-dioxo-9,10-dihydroanthracen-2-yl)methyl carbamimidothioate hydrochloride (compound 3) are depicted in Figure 1A. These derivatives were synthesized as previously reported [22]. Briefly, compound 1 was synthesized by treating 2,3-bis(bromomethyl)quinoxaline with selenourea (molar ratio 1:1.1) in absolute ethanol at room temperature for 2 h, and compound 2 by treating 2,3-bis(bromomethyl)quinoxaline with thiourea in absolute ethanol for 3.5 h at reflux. Compound 3 was obtained from 2-(chloromethyl)anthraquinone and thiourea (1:1.1 molar ratio) in absolute ethanol, stirring for 3 h at reflux. The purity of compounds was ≥ 99%.

### 2.2. Compounds 1 and 3 Reduced the Viability of Different Melanoma Cancer Cells

All the compounds were tested against a melanoma cell line (1205Lu) for their effect on cancer cell viability. The MTT (3-(4,5-dimethylthiazol-2-yl)-2,5-diphenyltetrazolium bromide) assay was performed in order to measure cancer cell viability, as previously described [22]. The anti-cancer effect of each agent was tested at seven different concentrations between 0.1 and 50 µM and three time points (24, 48, and 72 h). As shown in Figure 1B, compounds 1 and 3 potently inhibited the cancer cell viability of melanoma cells, while the isosteric sulfur analog (compound 2) of 1 was essentially ineffective up to the maximum concentration (50 µM) used. These results were in accordance with many of our previous reports [14,16,22,23,24] where isosteric replacement of sulfur by selenium in a small molecule significantly enhanced the anticancer activity. Therefore, compounds 1 and 3 were further screened against a panel of four melanoma cancer cell lines (WM2664, A375M, UACC903, and CHL-1) using the MTT assay. We compared the results with those obtained with PLX-4032, a B-RAF kinase inhibitor used currently in the clinic for melanoma treatment. The results are summarized in Table 1, expressed as IC_50_, the concentration that produces 50 % of growth inhibition.

Compound 1 was more cytotoxic across all cell lines compared to compound 3 and PLX-4032 (Figure 1, Table 1). Interestingly, while PLX-4032 was more effective when the B-RAF^V600E^ mutation was present, compounds 1 and 3 also reduced cell viability in the B-RAF wild-type cell line CHL-1, compound 1 being the most potent. It should be noted that PLX-4032 showed no effect at 24 h in four out of the five lines tested, whereas both compounds 1 and 3 produced a reduction of cell viability in the five cell lines at that time point (Figure 1, Table 1). Compound 1 exhibited an IC_50_ lower than 4 µM in all the cell lines tested at 24 h. Furthermore, this compound was more potent than the reference drug in all cell lines at the three time points, with the exception of A375M cell line after 72 h of treatment. Overall, the isoselenourea derivative (compound 1) exhibited much higher potency than the isothiourea analog (compound 3) and, therefore, we selected compound 1 for further in vitro efficacy and mechanism of action elucidation.

### 2.3. Compound 1 Suppressed the Proliferative Ability of Melanoma Cells (1205Lu and UACC903)

**Figure 2:**
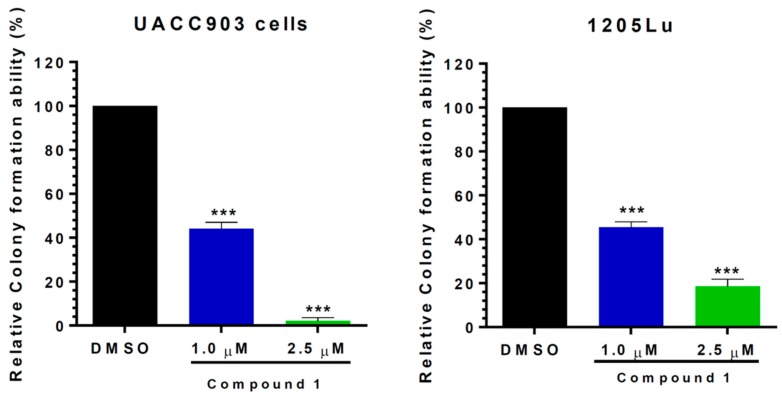
Compound 1 dramatically reduced the colony formation ability of melanoma cells. 1205Lu (**A**) and UACC903 (**B**) cells were treated with 1 or 2.5 µM of compound 1 or dimethyl sulfoxide (DMSO) (control) for 24 h. Subsequently, 500 cells were re-seeded with free compound 1 or DMSO medium for 14 days and stained with 0.5% alcoholic crystal violet. The results are expressed as mean ± SD. *** *p* < 0.001.

In order to evaluate the long-term effects of compound 1 on melanoma cells, a colony formation assay was employed. For this assay, the cells were treated with either compound 1 or dimethyl sulfoxide (DMSO, control) for 24 h, and then 500 live cells were counted and re-seeded in new plates in the absence of compound 1 or DMSO and allowed to form colonies over a period of 14 days. As shown in Figure 2, compound 1 effectively inhibited the colony formation ability of both 1205Lu and UACC903 melanoma cell lines. At the dose of 1 µM, the ability of melanoma cells to form colonies was reduced to less than 50%, and when cells were treated with 2.5 µM, this percentage was dramatically reduced at levels lower than 20% and 5% for 1205Lu and UACC903 cells, respectively. These studies clearly indicate that the effects of compound 1 on the inhibition of melanoma cells replication are long-lasting.

### 2.4. Compound 1 Increased Melanoma Cell Death in Vitro

**Figure 3:**
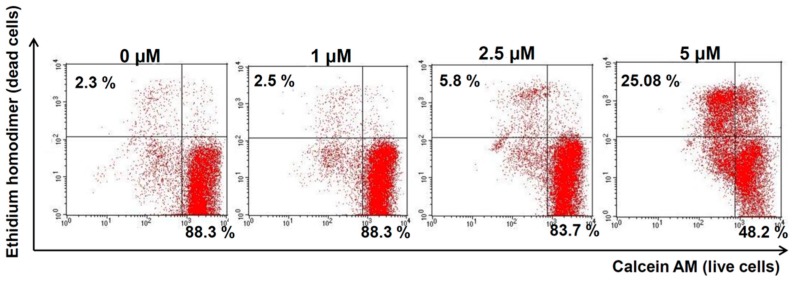
Compound 1 induced cell death in melanoma cells. 1205Lu cells were incubated with 1, 2.5, or 5 µM of compound 1 or DMSO (control) for 24 h and stained with ethidium homodimer and calcein AM. Live and dead cells were quantified by flow cytometry.

In order to study whether the reduction of cell viability caused by compound 1 was due to cell death and not cell growth inhibition, 1205Lu cells were subjected to the Live-and-Dead assay. As shown in Figure 3, compound 1 increased the number of cells positive for ethidium homodimer staining (dead cells, upper left quadrant) and reduced the cells stained with calcein AM (live cells, lower right quadrant) compared to control cells. After treatment with 1 µM compound 1, no difference was observed between control and treated cells. However, when the dose of compound 1 was increased to 5 µM, the percentage of dead cells increased dramatically up to 25 %. These results suggest that compound 1 was able to induce cell death in vitro in melanoma cells.

### 2.5. Compound 1 Induced Apoptosis in Melanoma Cells

With the aim of investigating whether the increase in cell death induced by compound 1 was due to apoptosis induction, the Muse^TM^ Annexin V & Dead Cell assay was carried out. Annexin V was employed in this assay to detect the externalization of phosphatidylserine to the cell surface, a process occurring in apoptosis but not in necrosis [25]. A dead cell marker (7-ADD) was also included in the kit as an indicator of cell membrane structural integrity. Therefore, cells negative for both markers (lower left quadrant) were healthy cells, cells positive for Annexin V only (lower right quadrant) were in early apoptosis, and cells positive for both Annexin V and 7-ADD were undergoing apoptotic death (upper right quadrant). Cells positive for 7-ADD only were undergoing necrosis (upper left quadrant).

**Figure 4:**
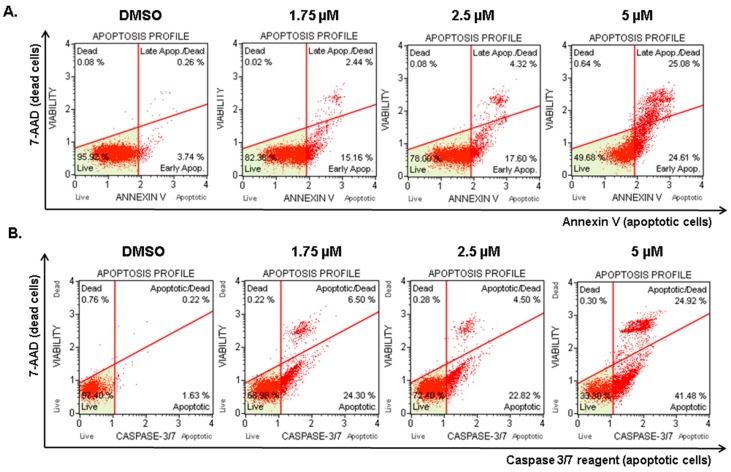
Compound 1 induced apoptotic cell death. After 24 h of incubation with the indicated concentration of compound 1 or DMSO (control), the apoptotic status of 1205Lu cells was analyzed using the Muse^TM^ Annexin V & Dead Cell Kit according to the manufacturer’s instructions. (**A**) Analogous independent experiments were analyzed with Muse^TM^ Caspase 3/7 Kit to confirm the results. (**B**) The results of both experiments were analyzed by flow cytometry.

Compound 1 was tested at three concentrations: 1.75, 2.5, and 5 µM. The dose of 1 µM was not tested because we observed no significant effect at this dose in the previous assay. As shown in Figure 4A, after the treatment with compound 1 at 1.75 µM concentration, 15% of cells were in early apoptosis (lower right quadrant). At 5 µM of compound 1, less than 50% of cells were healthy cells and 25% of cells died by apoptosis (upper right quadrant). Less than 1% of cells died without externalization of phosphatidylserine (upper left quadrant), indicating that compound 1 induced cell death through apoptosis.

In order to confirm these results, the Muse^TM^ Caspase-3/7 kit was also employed. The kit includes a reagent with a DNA binding dye. In non-apoptotic cells, this reagent is linked to an effector caspase recognition sequence which does not bind to DNA. However, when caspases are active, the dye is released by caspase cleavage and translocated to the nucleus, where it binds to DNA, producing high fluorescence. This kit also includes the dead cell marker 7-ADD. Hence, cells negative for both dyes were healthy cells (lower left quadrant), cells only positive for Caspase 3/7 reagent (lower right quadrant) were supposed to be in early apoptosis, cells positive for both dyes (upper right quadrant) were undergoing apoptotic death, and cells only positive for 7-ADD (upper left quadrant) were undergoing caspase-independent death. The results (Figure 4B) showed the same tendency observed in the Annexin V assay. The doses of 1.75 and 2.5 µM increased the amount of early apoptotic cells (lower right quadrant), and, after treatment with 5 µM compound 1, more than 60% of the cells were positive for caspase 3/7 activity (lower and upper right quadrants). Less than 0.5% of cells died through caspase-independent mechanisms. Overall, our results confirmed that compound 1 is a potent apoptosis inducer. 

### 2.6. Compound 1 did not Inhibit the Phosphorylation of Akt and ERK1/2

**Figure 5:**
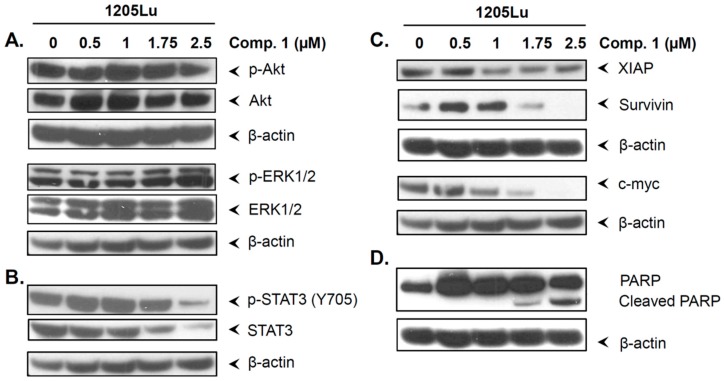
Compound 1 downregulated STAT3 and its downstream target proteins expression. 1205Lu cells were incubated with either DMSO (control) or compound 1 for 24 h. Whole cell lysates were subjected to western blot analysis. Expression of different proteins related to STAT3 and its downstream targets (**A**–**D**) were monitored. ß-actin was used as a loading control.

The dysregulated expression of Akt and ERK1/2 is associated with cell proliferation and melanoma cell survival [26,27]. Hence, we decided to evaluate whether compound 1 affected the expression and phosphorylation status of these proteins. Interestingly, as shown in Figure 5A, compound 1 did not affect the expression or phosphorylation of Akt or ERK1/2. These observations suggest that the cell inhibitory response to compound 1 is not mediated through the inhibition of Akt and ERK1/2; therefore, other pathways might be implicated.

### 2.7. Compound 1 Inhibited STAT3 and Related Proteins Expression

To further examine the mechanism of action of compound 1, its effects on proteins implicated in cancer survival and metastasis were studied. STAT3 is highly upregulated in melanoma, and its activation by phosphorylation contributes to cancer progression and survival [12]. Hence, we decided to establish whether compound 1 affected the expression and phosphorylation status of the transcription factor STAT3. As shown in Figure 5B, compound 1 reduced protein levels and phosphorylation of STAT3. At the dose of 2.5 µM, the expression and phosphorylation of STAT3 were dramatically reduced. 

STAT3 controls the expression of proteins involved in proliferation, survival, and metastasis formation, such as XIAP, survivin, and c-myc [28,29,30]. As observed in Figure 5C, compound 1 also downregulated these proteins at the doses at which STAT3 inhibition was observed. In addition, the inhibition of these anti-apoptotic proteins was accompanied by PARP (poly(ADP-ribose) polymerase) cleavage (Figure 5D), which is traditionally employed as a marker of apoptosis [31]. These results suggest that compound 1 has the ability to inhibit STAT3 signaling pathway. Moreover, the induction of apoptosis and cell proliferation inhibitory response of compound 1 may be mediated by STAT3 inhibition.

### 2.8. Compound 1 Induced the Cell Cycle Inhibitor p21

**Figure 6:**
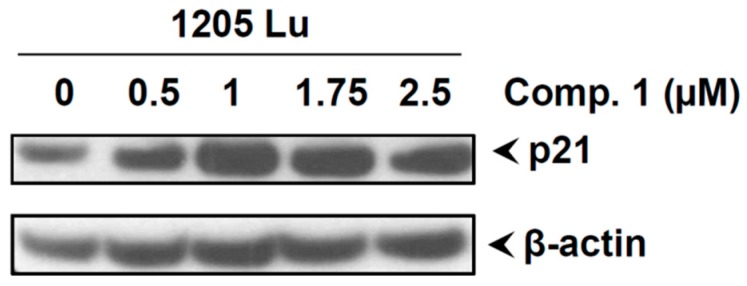
Compound 1 induced p21 expression. After 24 h of treatment with either compound 1 or DMSO (control), 1205Lu cells were collected, and the expression of p21 was analyzed by western blot analysis. ß-actin was used as a loading control.

Recent studies have shown a correlation between the cell cycle inhibitor p21 and transcription factor STAT3 [32]. These studies revealed that p21 is part of a feedback network controlling the down-modulation of STAT activity [32]. Thus, we sought to investigate whether compound 1 activated the expression of this cell cycle inhibitor. As shown in Figure 6, compound 1 induced the expression of p21. These results suggest that the cell inhibitory activities of compound 1 may be mediated by activation of p21. 

### 2.9. Compound 1 Inhibited the Phosphorylation of STAT3, Increased the Expression of p21, and Induced Apoptotic Cell Death in Different Melanoma Cell Lines

**Figure 7:**
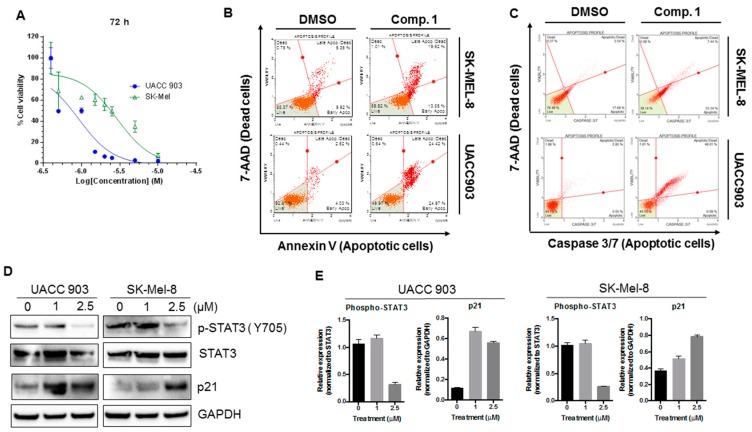
Compound 1 inhibited the phosphorylation of STAT3, increased the expression of p21, and induced apoptotic cell death in UACC 903 and Sk-Mel-8 melanoma cell lines. (**A**) Cell viability (MTT) results for compound 1 in two different melanoma cell lines at 72 h. The graphs were obtained by performing non-linear regression analysis using variable slope. Error bars represent mean ± SD. (**B**,**C**) Human melanoma cells UACC 903 and SK-Mel-8 were treated with compound 1 (2.5 μM) for 24 h, and an apoptotic assay was performed using the Muse^TM^ Annexin V & Dead Cell (**B**) and Caspase 3/7 Kit (**C**) according to the manufacturer’s instructions. (**D**) Melanoma cells (UACC 903 and SK-Mel-8) cells were treated with the mentioned concentrations of Compound 1. After 24 h, cells were collected and lysed, and the expression of p-STAT3, STAT3, and p21 was analyzed by western blot. GAPDH was used as a loading control; (**E**) Quantification of protein expression was performed using Image J software, and graphs represent the relative expressions of proteins. The relative expressions were determined using either STAT3 or GAPDH as mentioned.

The data above demonstrate that compound 1 effectively decreased p-STAT3 levels and increased the expression of p21 and apoptotic cell death in 1205LU cells. However, to rule out cell line-dependent effects of compound 1, we tested all the above biological activities of compound 1 in two additional melanoma cell lines (UACC903 and SK-Mel-8). As shown in Figure 7A, compound 1 inhibited cell proliferation of both melanoma cell lines, with UACC903 cells being more sensitive than SK-Mel-8. As suggested by our Annexin V and Caspase 3/7 activity assay (Figure 7B,C), compound 1 induced apoptotic cell death. Additionally, compound 1 also decreased the phosphorylation of STAT3 (Figure 7D,E) and induced p21 expression similar in both cell lines, to what observed in 1205Lu cells. Hence, the biological activities of compound 1 were consistent across a variety of human melanoma cell lines. 

### 2.10. Docking

**Figure 8:**
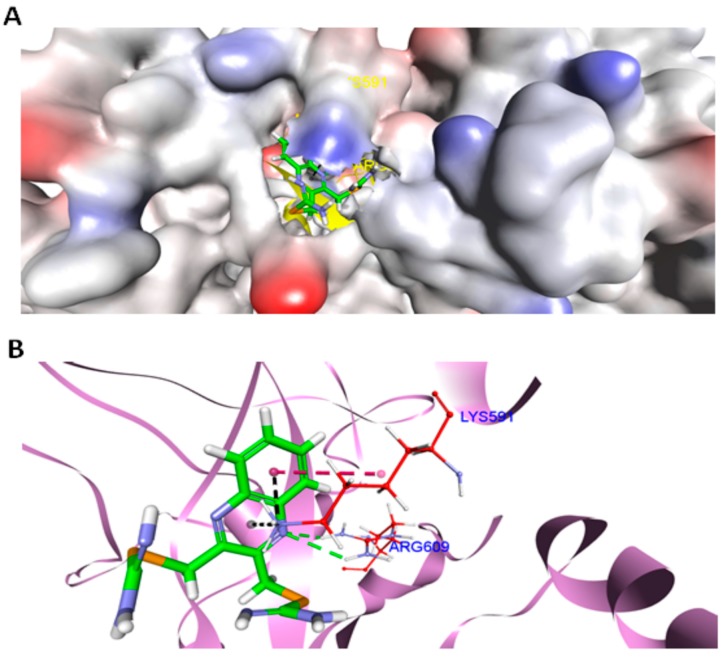
Docked complex of compound 1 with STAT3. (**A**) The surface representation of STAT3 showing deep penetration of compound 1 into the SH2 domain of STAT3 (pdb id: 1BG1), generated by DS (Accelrys). (**B**) Docked conformation of compound 1 in the SH2 domain of STAT3 (pdb id: 1BG1), viewed by DS (Accelrys). Carbon atoms of compound 1 (in sticks format) are colored green. The interacting amino acids of the SH2 domain of STAT3 are colored in red. All other amino acids of STAT3 are depicted in violet (flat ribbon format). Hydrogen bonds are shown in green, electrostatic interactions in black, and hydrophobic interactions are shown as magenta dotted lines.

In order to substantiate our experimental findings and to investigate the binding propensities of compound 1 to the SH2 domain of STAT3, docking simulations were employed. The molecular structure [33] of STAT3 (pdb id: 1BG1, resolution 2.25 Å) was retrieved from the protein data bank (www.rcsb.org). CDocker [34], a CHARMm force field-based algorithm embedded in DS version 4.0 (Accelrys; San Diego, CA, USA), was used to flexibly dock compound 1 within the conservative SH2 domain of STAT3. The docking analysis revealed favorable binding of compound 1 with STAT3, based on the computed scoring function (CDocker energy = −23.8 kcal/mol), where the most negative value of CDocker energy indicates good binding affinity of a ligand for a protein. The visualization of the complex (Figure 8A) further revealed that compound 1 penetrated deep inside the STAT3 cavity and settled well by establishing a network of hydrogen bonding and electrostatic forces with the protein. Specifically, compound 1 utilized its nitrogen atom (proton acceptor) of the quinoxaline moiety to form two strong concurrent hydrogen bonds (2.2 Å, 2.8 Å) with the amine (proton donor) functionalities of Arg609 (Figure 8B). This amino acid contributed significantly to STAT3 and SH2 peptide binding, as the mutation of Arg609 has been reported to abolish the peptide-binding ability of this domain [35,36]. Several anticancer agents targeting the same amino acid residue (Arg609) in STAT3 SH2 domain have already been documented in the literature [37,38]. Additionally, a hydrophobic interaction and two electrostatic forces (cation-type) between the –NH_3_ group of Lys591 and the aromatic network of compound 1 also facilitated locking its conformation in the binding domain of STAT3. Overall, the docking results revealed compound 1 as a good inhibitor of STAT3 protein and supported our experimental observations.

## 3. Discussion

In this work, we tested the effects of an isoselenourea derivative and an isothiourea compound on cell viability in a panel of five melanoma cell lines bearing different mutations. The sulfur analog of compound 1 (compound 2) was inactive at the studied doses. This was in accordance with a previous report showing the isoselenourea derivative PBISe, an isosteric selenium analog of PBIT, to be over 10-fold more effective in inhibiting the viability of melanoma cells [19]. However, our recent report [22] also indicates that replacing the isothiourea functionality by an isoselenourea may not always lead to a more potent compound, suggesting that the potency depends on the overall structure of the molecule. For example, the isothiourea analog compound 3 was more effective than its corresponding isoselenourea analog [22]. Compounds 1 and 3 were active against both B-RAF mutant and wild-type (CHL-1) cells lines, whereas PLX-4032 is only effective against the altered form [39]. In addition, our compounds were also more effective in reducing cell viability at 24 h in all the tested cell lines, compound 1 being at least 10-fold more potent than PLX-4032. In general, as reported in previous works [14,22,23], the selenium-containing derivative was much more active than the isothiourea one in reducing cancer cell growth.

The results from Annexin V and Caspase3/7 assays, as well as the observation of cleaved PARP in western blot, indicated that compound 1 induced apoptotic cell death. Apoptosis is a typical mechanism for selenium compounds to induce cell death [13]. However, the underlying mechanism of apoptosis induction could be remarkably different between distinct selenium-containing small molecules. Activation of caspases, modulation of anti-apoptotic proteins, alteration of oxidative stress-related proteins, cell cycle arrest, or kinases regulation are some of the described mechanisms associated with apoptosis [40]. This fact could explain the different mechanism of action between PBISe and compound 1. Although PBISe inhibited the phosphorylation and total protein levels of Akt in melanoma cells [41], compound 1 did not regulate this protein. We conclude that compound 1 is not effective against either Akt or ERK1/2 and indeed we found that compound 1 inhibits the expression of STAT3. In silico docking simulations conducted on compound 1 using STAT3 as a molecular target also suggested the compound to be a good inhibitor for this target protein. Hydrogen bonding and electrostatic and hydrophobic forces were found to be accountable for their host–guest relationship. Moreover, just like other known STAT3 inhibitors, compound 1 also interacted with Arg609, an essential amino acid for STAT3 function, suggesting its anti-melanoma activity may well be through inhibition of STAT3, in agreement with the experimental results. 

STAT3 is a member of the signal-transducer-and-activators-of-transcription family. When phosphorylated, STAT3 dimerizes and translocates to the nucleus, where it modulates the transcription of genes involved in cell survival and proliferation [42]. Constitutive activation of STAT3 has been reported in several tumor types and has been proved to be involved in proliferation, survival, inflammation, invasion, metastasis, and angiogenesis [7]. Several in vitro and in vivo studies have demonstrated that the inhibition of STAT3 leads to tumor growth inhibition [5]. Thus, STAT3 is considered a valid therapeutic target for cancer therapy. STAT3 also has an important role in melanoma development and survival. This protein was found to be constitutively activated in numerous melanoma cell lines and tumor specimens [5]. Furthermore, inhibition of STAT3 signaling led to apoptosis of melanoma cells. Besides, STAT3 upregulation has been associated with acquired resistances to PLX-4032. Melanoma cells resistant to PLX-4032 showed increased STAT3 pathway activity [43], and in vitro silencing of this signaling inhibited the growth of cells resistant to PLX-4032 [11]. Moreover, a combination treatment with WP1066 (a STAT3 inhibitor) and PLX-4032 resulted in more significant growth inhibition of both resistant and sensitive cells to PLX-4032. The downregulation of other proliferative and anti-apoptotic proteins which are under transcriptional regulation of STAT3, such as XIAP, survivin, and c-myc, supports that compound 1 induces apoptosis through STAT3 inhibition. A previous study also reported a relationship between selenium-containing compounds and STAT3 inhibition [44]. The studies by Zuazo et al. demonstrated that imidoselenocarbamate derivatives blocked hypoxia-induced STAT3 phosphorylation [44]. 

## 4. Materials and Methods

### 4.1. Chemistry

All the chemicals were obtained from Sigma Aldrich (Alcobendas, Madrid, Spain) and Acros Organics (Janssen Pharmaceuticalaan, Geel, Belgium). Quinoxaline-2,3-diylbis(methylene)dicarbamimidoselenoate dihydrobromide (compound 1), quinoxaline-2,3-diylbis(methylene)dicarbamimidothioate dihydrobromide (compound 2), and (9,10-dioxo-9,10-dihydroanthracen-2-yl)methyl carbamimidothioate hydrochloride (compound 3) were synthesized and characterized as previously described [22]. Briefly, the corresponding alkyl halide (1 mmol) was added to a mixture of selenourea (2.2 mmol; compound 1) or thiourea (2.2 mmol for compound 2 and 1.1. mmol for 3) in absolute ethanol (20 mL). The mixture was stirred 2 h at r.t. for compound 1 or 3 hat reflux for 2 and 3. The precipitate was filtered and washed with 50 mL of ether (1 and 2) or with dichloromethane and ether (3).

### 4.2. Reagents and Antibodies

All the chemicals were obtained from Sigma Aldrich (Alcobendas, Madrid, Spain) and Acros Organics (Janssen Pharmaceuticalaan, Geel, Belgium). Quinoxaline-2,3-diylbis(methylene)dicarbamimidoselenoate dihydrobromide (compound 1), quinoxaline-2,3-diylbis(methylene)dicarbamimidothioate dihydrobromide (compound 2) and (9,10-dioxo-9,10-dihydroanthracen-2-yl)methyl carbamimidothioate hydrochloride (compound 3) were synthesized and characterized as previously described [22]. Briefly, the corresponding alkyl halide (1 mmol) was added to a mixture of selenourea (2.2 mmol; compound 1) or thiourea (2.2 mmol for compound 2 and 1.1. mmol for 3) in absolute ethanol (20 mL). The mixture was stirred 2 h at r.t. for compound 1, or 3 h at reflux for compounds 2 and 3. The precipitate was filtered and washed with 50 mL of ether (1 and 2) or with dichloromethane and ether (3). Rabbit anti-phospho-Akt (Cat # 4060), rabbit anti-Akt (Cat # 4685), rabbit anti-phospho-STAT3 (Cat # 9145), rabbit anti-STAT3 (Cat # 12640), rabbit anti-phospho-p44/42 MAPK (Erk1/2) (Cat # 4370), rabbit anti-p44/42 MAPK (Erk1/2) (Cat # 4695), rabbit anti-XIAP (Cat # 14334), rabbit anti-survivin (Cat # 2803), rabbit anti-c-Myc (Cat #9402), rabbit anti-p21waf1/Cip1 (Cat # 2947), and rabbit anti-PARP (Cat # 9542) were obtained from Cell Signaling (Danvers, MA). Antibodies against GAPDH, β-actin, goat anti-rabbit, and goat anti-mouse horseradish peroxidase conjugates, and MTT were purchased from Sigma-Aldrich (St. Louis, MO, USA).

### 4.3. Cell Culture

Human melanoma cell lines were grown in DMEM medium supplemented with 10 % fetal bovine serum (FBS) and 100 units/mL of penicillin and streptomycin (Corning; Corning, NY, USA). The cells were maintained at 37 °C and 5% CO_2_. 

### 4.4. Cell Viability

A total of 3000 cells/well were grown in 96-well plates for 12 h and then treated with either DMSO (control) or increasing concentrations (0.5–50 µM) of compound 1, 3, or PLX-4032 for 24, 48, and 72 h. Three hours before the termination point, 20 µL of MTT were added to measure cellular viability. The resultant formazan crystals were dissolved in 50 µL of DMSO, and absorbance was measured at 570 nm and 630 nm wavelengths. IC_50_ values were calculated using GraphPad Prism version 6.01.

### 4.5. Colony Formation Assay

Melanoma cells (UACC903 and 1205Lu) were treated with DMSO or compound 1 (1 and 2.5 µM) for 24 h. The cells were then trypsinized and counted, and 500 live cells were seeded in new 10 cm tissue culture plates separated by treatments. The cells were allowed to form colonies for 14 days in DMEM without compound 1 or DMSO in a 5% CO_2_ incubator. After 14 days, the medium was removed, and the formed colonies were stained with 0.5% alcoholic crystal violet. The percentage plating efficiency (PE) was calculated by using the following formula:

(1) $$\%PE=\frac{number\;of\;colonies}{number\;of\;cells\;plated}\times 100$$

The results correspond to the mean ± SD of three independent experiments. Differences between control and treated cells were determined by one-way ANOVA tests, using GraphPad Prism version 6.01.

### 4.6. Live-and-Dead Assay

The LIVE/DEAD viability/cytotoxicity kit for mammalian cells (Molecular Probes, Invitrogen; Carlsbad, CA, USA) was employed for this experiment. A total of 7.5 × 10^5^ 1205Lu cells/well were plated in a 6-well plate and treated with DMSO (control) or increasing amounts of compound 1. After 24 h of incubation, both floating and attached cells were collected and re-suspended in PBS. Further, cells were stained with a calcein AM and ethidium homodimer solution according to the manufacturer’s instructions. The number of live and dead cells was determined by flow cytometry (BD FACSCalibur; Heidelberg, Germany).

### 4.7. Annexin V Assay

1205Lu, UACC903, and SK-Mel-8 (4 × 10^5^ cells/well) were pleated and treated with DMSO (control) or compound 1 at the given concentrations. After 24 h of treatment, both floating and attached cells were collected and stained with the Muse^TM^ Annexin V & Dead Cell Reagent (Millipore; Bedford, MA, USA) for 20 min at room temperature in the dark. The results were collected by Muse^TM^ Cell Analyzer (Millipore).

### 4.8. Caspase-3/7 Assay

The Muse^TM^ Caspase 3/7 kit (Millipore) was employed for this experiment according to the manufacturer’s instructions and our previously published method [22]. 1205Lu, UACC903, and Sk-Mel-8 cells were seeded at a density of 4 × 10^5^ cells/well and treated with DMSO or the indicated amounts of compounds 1 and 3 for 24 h. Next, both floating and attached cells were collected, out of which 50 µL of cells were stained with 5 µL of Muse^TM^ Caspase-3/7 working solution and incubated for 30 min at 37 °C with 5 % CO_2_. After incubation, 150 µL of Muse^TM^ Caspase 7-AAD working solution were added to each sample, and the samples were incubated in the dark for 5 min more. The samples were analyzed by Muse^TM^ Cell Analyzer.

### 4.9. Western Blotting

1205Lu, UACC903, and SK-Mel-8 cells (7 × 10^5^ cells/well) were plated in 6-well plates and treated with compound 1 or DMSO for 24 h. The cells were then collected, and whole cell lysates were prepared in RIPA buffer (Thermo Scientific #89900; Rockford, IL, USA) supplemented with 1% phosphatase inhibitor cocktail 2 (Sigma #P5726-5ML), 1% protease inhibitor (Complete mini, Roche #11836170001; Branchburg, NJ, USA), and 0.5 % of 200 mM phenylmethanesulfonyl fluoride (PMSF) (Sigma #P7626-250 mg). The cell lysates were spun at 15,000× *g* for 10 min to remove any insoluble cell debris. The resultant supernatants were collected and stored at −80 °C until use. The whole cell lysates were resolved by SDS-PAGE. The proteins were transferred to Immobilon^®^-P PVDF membranes (Millipore # IPVH304F0) and blotted with the indicated antibody overnight at 4 °C. The dilution for primary antibodies was 1:1000, with the exception of p-STAT3 (1:2000), STAT3 (1:200), and β-actin (1:3000). Further, the membranes were incubated with the corresponding peroxidase-linked secondary antibodies (dilution 1:3000) for 1–4 h at room temperature. The antibodies were detected by an enhanced chemiluminescence reagent (Thermo Scientific #1856135 and #1856136).

### 4.10. Docking Methodology

Different 3D conformations of compound 1 were generated and energetically minimized using the “Generate Conformations” tool in Discovery Studio (DS) 4.0 client (Accelrys). The lowest energetic conformation thus obtained was subjected to the “Prepare Ligands” module to generate its isomers at physiological pH. The CHARMm force field was employed to develop the partial atomic charges on each atom of the isomer. The isomer with the lowest CHARMm energy was used for the docking study.

The X-ray co-ordinates of STAT3 (pdb id: 1BG1, resolution 2.25 Å) were retrieved from the protein data bank (www.rcsb.org). The “Prepare Protein” tool in DS was used to add missing atoms/chains and remove water molecules in the protein structure. The “Prepare Protein” algorithm was employed to protonate amino acid residues according to the physiological conditions. Prior to docking, a binding sphere covering the SH2 domain of STAT3 was generated. CDOCKER [34], a grid-based docking program, was used to dock compound 1 in the SH2 domain, considering the default parameters. The most favorable pose of compound 1 was identified based on the CDOCKER energy (-CDE).

## 5. Conclusions

In conclusion, compound 1, a quinoxaline-isoselenourea, showed promising efficacy in vitro against melanoma cells. Compound 1 was more effective than the isothiourea derivative 3 and the reference drug PLX-4032, especially at short times of treatment (24 h). As demonstrated by the colony formation assay, the effects of compound 1 in inhibiting cell proliferation were long-lasting. Overall, we demonstrated for the first time that the cell growth inhibitory response of compound 1 may be mediated through the STAT3 pathway as it does not inhibit other pro-survival signaling pathways such as Akt and ERK1/2. On account of the fact that compound 1 inhibits STAT3, it could also be employed in combination therapy with PLX-4032 to overcome acquired resistance to the latter. Interestingly, the response to compound 1 was not dependent on the mutation status of BRAF. Only about half of all melanomas have a mutation in the BRAF gene, and, therefore, compound 1, targeting both BRAF-mutant and WT melanoma cells, may have a broader clinical impact. However, more preclinical studies examining in vivo efficacy, Absorption, Distribution, Metabolism and Excretion (ADME), toxicity, and mechanism of action are required to determine the therapeutic properties and future clinical potential of compound 1.

## Figures and Tables

**Table 1:** Effects on cell viability of compounds 1 and 3 and PLX-4032 in five different melanoma cell lines. The results are expressed as mean values for IC_50_ with their respective standard deviation (± SD).

Comp.	Time (h)	Cell Lines				
		WM2664	1205Lu	A375M	UACC903	CHL-1
**1**	24	1.8 ± 0.4	2.6 ± 0.3	2.6 ± 1.0	3.8 ± 1.6	2.9 ± 0.4
	48	1.1 ± 0.3	1.9 ± 0.3	1.9 ± 0.1	0.8 ± 0.2	3.1 ± 0.4
	72	2.0 ± 0.2	2.0 ± 0.6	1.1 ± 0.2	0.3 ± 0.6	2.5 ± 0.2
**3**	24	32.7 ± 3.6	33.7 ± 10.1	38.7 ± 1.0	32.6 ± 1.9	29.3 ± 10.6
	48	21.0 ± 2.9	11.3 ± 3.0	21.3 ± 0.8	24.7 ± 2.3	12.3 ± 2.2
	72	22.8 ± 7.0	8.3 ± 0.9	8.1 ± 4.2	22.3 ± 5.6	13.6 ± 1.7
**PLX-4032**	24	>50.0	>50.0	>50.0	38.5 ± 7.2	>50.0
	48	32.1 ± 14.2	31.4 ± 8.0	3.5 ± 1.1	10.3 ± 2.6	21.2 ± 3.4
	72	3.6 ± 1.5	9.3 ± 3.4	0.4 ± 0.3	17.9 ± 1.3	12.2 ± 1.5

## Abbreviations

AKT	Protein kinase B
c-myc	Avian myelocytomatosis virus oncogene cellular homolog
ERK1/2	Extracellular signal-regulated kinases 1 and 2
FBS	Fetal bovine serum
MAPK	Mitogen-activated protein kinase
MMP	Matrix metalloproteinase
MTT	3-(4,5-dimethylthiazol-2-yl)-2,5-diphenyltetrazolium bromide
PARP	Poly(ADP-ribose)polymerase
PBISe	*Se*,*Se’*-1,4-phenylenebis(1,2-ethanediyl)bisisoselenourea
PBIT	*S*,*S’*-1,4-phenylenebis(1,2-ethanediyl)bisisothiourea
PMSF	Phenylmethanesulfonyl fluoride
STAT3	Signal transducer and activator of transcription 3
XIAP	X-linked inhibitor of apoptosis protein
